# N-carbamylglutamate supplementation improves laying performance of layers by regulating hypothalamic-pituitary-ovarian axis

**DOI:** 10.3389/fvets.2025.1668137

**Published:** 2025-10-02

**Authors:** Xiao-Bing Peng, Qing-Yue Wang, Yan Zhang, Na Liu, Wei Ma, Chun-Qiang Wang

**Affiliations:** College of Animal Science and Veterinary Medicine, Jinzhou Medical University, Jinzhou, China

**Keywords:** layer, transcriptomics analysis, hypothalamic-pituitary-ovarian axis, egg production performance, N-carbamylglutamate

## Abstract

This study aimed to investigate the effects of dietary supplementation of N-carbamylglutamate (NCG) on the laying performance of layers and explore the underlying endocrine mechanism involving the hypothalamic-pituitary-ovarian (HPO) axis. Ninety-six 12-week-old layers of the Zhuanghe Dagu breed were divided into two groups: CON and TRT, with four replicates and 12 birds per replicate. The experimental period lasted 24 weeks, during which the CON group received a basal diet while the TRT group received a basal diet supplemented with 0.12% NCG. The results showed that NCG supplementation in the diet resulted in an increase in the egg production rate and an advancement in the timing of egg-laying compared to the CON group. To gain insights into the underlying molecular mechanisms, transcriptomics analysis was conducted on the hypothalamus, pituitary, and ovary. Differential gene expression analysis identified 156 differentially expressed genes (DEGs) in the hypothalamus, 208 DEGs in the pituitary, and 229 DEGs in the ovary. Pathway analysis revealed that these DEGs were enriched in 2 pathways in the hypothalamus, 8 pathways in the pituitary, and 9 pathways in the ovary, all of which are related to reproduction. Of particular interest, the expression of specific genes involved in the HPO axis, such as *FSHB* and *GNRH1* in the hypothalamus, *DHH* and *GNRHR* in the pituitary, and *RSPO1*, *ZP3*, *GSTA3*, *C14orf39*, *HOXA10*, and *IRX5* in the ovary, was significantly regulated by NCG supplementation. These findings were further validated by quantitative real-time polymerase chain reaction, which confirmed the expression profiles of the aforementioned genes observed in the RNA-seq results. Overall, these findings provide valuable insights into the endocrine mechanisms underlying the improvement of laying performance in layers through NCG supplementation.

## 1 Introduction

Eggs are a globally consumed high-quality protein source free from religious restrictions. The egg production rate is one of the key indicators for evaluating the performance of laying hens. Animal nutritionists have long been working to develop strategies to improve egg production efficiency, meeting the people’s growing demand for high-quality eggs. Key parameters such as the age at first egg-laying, egg production rate, and egg weight are of particular interest as they have significant economic implications for poultry farming (1). From a physiological perspective, the hypothalamic-pituitary-ovarian (HPO) axis play crucial roles in processing signals and precisely regulating reproductive activities in layers, the production of eggs is directly regulated by it. (2, 3).

N-Carbamylglutamate (NCG) has been shown to effectively stimulate the synthesis of endogenous arginine (4). When added to the diet, NCG has been found to have numerous positive effects on the growth and reproductive performance of birds (5). In a previous study, it was observed that feeding roosters a diet supplemented with NCG increased circulating levels of reproductive hormones and enhanced the development of sexual organs (6). Furthermore, NCG supplementation has been reported to promote follicle development (7). In a recent study by Ma et al. (8), a transcriptomic analysis of the uterus in layers revealed that NCG supplementation improves production performance and enhances egg quality by regulating uterine function.

In avian species, the HPO axis plays a central role in regulating reproductive processes. Specifically, the hypothalamus secretes gonadotropin-releasing hormone-I (GnRH-I), which stimulates the pituitary gland (adenohypophysis) to release follicle-stimulating hormone (FSH) and luteinizing hormone (LH). These hormones, in turn, promote ovarian secretion of estradiol (E2) and progesterone (P4), essential for ovulation. While previous studies have explored the molecular mechanisms underlying hen egg-laying performance, the differential gene expression and key signaling pathways governing this process across the HPO axis remain poorly understood. To address this gap, we investigated how dietary supplementation with NCG modulates egg-laying performance via the HPO axis. Our study combined phenotypic assessment of laying efficiency with transcriptomic profiling of the hypothalamus, pituitary, and ovaries to elucidate the regulatory mechanisms involved.

## 2 Materials and methods

The protocol of this study was proposed according to the ARRIVE guidelines^1^ for the reporting of animal experiments and was reviewed and approved by Jinzhou Medical University Animal Care and Use Committee. All experimental procedures and methods were approved by the Ethics Committee of Jinzhou Medical University and were conducted in accordance with the relevant guidelines established by the Ministry of Agriculture of the People’s Republic of China.

### 2.1 Experimental design

A total of 96 12-week-old Zhuanghe Dagu layers were utilized in a 24-week feeding trial. The birds were randomly assigned to two groups: CON and TRT, with each group consisting of 4 replicates, and 12 birds per replicate. The CON group received a basal diet, while the TRT group received a basal diet supplemented with 0.12% NCG. The basal diet was formulated based on Nutrient Requirements of Poultry provided by the National Academic Press (9) and was provided in mash form (Table 1). The NCG used in this study was obtained from Anhui Pusheng Pharmaceutical Co. Ltd., a commercial company. The dosage of NCG was determined based on our previous study conducted by Ma et al. (8).

**Table 1:** Composition and nutrient levels of the experimental basal diet, (%, as-fed basis).

Ingredients, %	
Corn	63.18
Soybean meal	25.60
Calcium bicarbonate	1.30
Limestone	8.70
NaCl	0.26
Baking soda	0.20
Vitamin and trace mineral premix^1^	0.50
Methionine	0.23
Lysine	0.03
Total	100.00
Analyzed composition, %	
Metabolizable energy, MJ/kg	11.03
Crude protein	16.50
Calcium	3.41
Phosphorus	0.32
Lysine	0.80
Methionine	0.51
Total sulfur amino acid	0.80
Tryptophan	0.18

1 Provided per kg of complete diet: Cu 20 mg, Fe 70 mg, Zn 70 mg, Se 0.5 mg, Vitamin A 7,000 IU, Vitamin D_3_ 2,500 IU, Vitamin E 30 mg, Vitamin K_3_ 1 mg, Vitamin B1 1.5 mg, Vitamin B2 4 mg, Vitamin B6 1.5 mg, Niacin 30 mg, folic acid 0.55 mg, D-pantothenic acid 10 mg, Vitamin B12 0.02 mg, biotin 0.16 mg, choline 400 mg.

The layers were housed in a well-ventilated room with programmable lighting and natural ventilation. They were reared in adjacent steel cages equipped with nipple drinkers, common trough feeders, and an egg collection plate. Throughout the experimental period, the average room temperature was maintained at 23 °C. The lighting program followed a schedule of 16 h of light and 8 h of darkness. All layers had ad libitum access to feed and water.

### 2.2 Sampling and measurements

#### 2.2.1 Production performance

The day of starting egg-laying was determined as the first day when at least 6 layers within a replicate began laying eggs (10).

From weeks 26 to 36 of age, the number and weight of eggs, as well as the feed intake, were recorded on a daily basis for each replicate. These data were used to calculate the average daily feed intake (ADFI) and egg production rate. The feed conversion ratio (FCR) was calculated by dividing the ADFI by the total egg weight.

On the final day of the study, all birds were euthanized via intravenous injection of 1 cc Euthasol. Tissue samples of the hypothalamus, pituitary, and ovarian were then collected from the carcasses, all the hypothalamus, pituitary labeled, and preserved in liquid nitrogen for subsequent experiments; regarding ovarian tissue, a random selection of 8 ovaries per group was used for follicle classification and measurement, the remaining ovaries were properly labeled and cryopreserved in liquid nitrogen for subsequent experimental procedures.

Follicles were classified according to established morphological and size-based criteria (11). The ovaries were placed in ice-cold PBS. Visible follicles (>1 mm diameter) were gently separated from stromal tissue using fine forceps under a stereomicroscope (Leica M80, 10× magnification). Follicle classification followed established standards: Small White Follicles (SWF, 1–2 mm), Large White Follicles (LWF, 2–5 mm), Small Yellow Follicles (SYF, 5–8 mm), Large Yellow Follicles (LYF, 8–12 mm), and Hierarchical Follicles (>12 mm).

#### 2.2.2 Transcriptomics analysis of tissues

In this study, 6 chickens were randomly selected in each group, and their hypothalamus, pituitary, and ovary were collected for study, approximately 0.2 g of hypothalamic, pituitary, and ovarian tissue was collected and used for total RNA extraction using Trizol reagent. The integrity of the RNA was assessed by agarose gel electrophoresis, and its purity was evaluated using a Nanodrop 2000 spectrophotometer. The concentration of RNA was accurately quantified using the Qubit 2.0 system, and RNA integrity was assessed using the Agilent 2100 Bioanalyzer. For each sample, a total of 3 μg of RNA was used as input material for RNA sample preparations. Sequencing libraries were generated using the NEBNext Ultra RNA Library Prep Kit for Illumina, following the manufacturer’s instructions. Index codes were added to attribute sequences to each sample. The quality of the libraries was assessed using the Agilent Bioanalyzer 2100 system.

The libraries were sequenced using the Illumina HiSeq 2500 platform. Quality control of the reads was performed using custom scripts. The raw data in Fastq format were processed using in-house Perl scripts, which involved removing reads containing adapters, poly-N sequences, and low-quality reads. Quality metrics such as Q20, Q30, and GC content were calculated for the clean data. All subsequent analyses were performed using the high-quality clean data. The paired-end sequencing strategy with PE 150 was employed in this study. The chicken genome sequence (version 90) was obtained from the genome website.^2^ The reference genome index was built using Hisat2 v2.0.5, and the paired-end clean reads were aligned to the reference genome using Hisat2 v2.0.5. The gene expression level was estimated using the fragments per kilobase of transcript per million fragments mapped method. Differential expression analysis between the groups was performed using the DESeq2 R package (version 1.16.1) based on the read count data. Functional annotation and pathway enrichment analysis were conducted using the Kyoto Encyclopedia of Genes and Genomes (KEGG) database.^3^ The statistical enrichment of differentially expressed genes in KEGG pathways was assessed using the ClusterProfiler R package.

#### 2.2.3 qRT-PCR verification

To validate the transcriptome sequencing data obtained through RNA-seq, the expression levels of specific genes, including *FSHB*, *GNRH1*, *POSTN* and *SCNN1B* in the hypothalamus, *POSTN*, *SCNN1B* and *GNRHR* in the pituitary, and *COL6A2*, *FN1 POSTN* and *SCNN1B* in the ovary, were measured using quantitative real-time PCR. Total RNA was extracted from the tissue samples using a total RNA reverse transcriptase kit (Takara, Dalian, China). Subsequently, cDNA synthesis was performed. Real-time PCR was conducted on an ABI 7500 Fast Real-Time PCR system using SYBR premix Ex TaqTM II (Takara). The optimized cycling conditions consisted of an initial denaturation step at 94 °C for 5 min, followed by 45 cycles of denaturation at 94 °C for 15 s, and annealing/extension at 55 °C for 15 s. Each sample was tested in triplicate to ensure the reliability of the results. The relative expression levels were determined using the 2^−ΔΔCt^ method (12), with β-actin serving as the internal control for data normalization. The primer sequences used for amplifying the target genes were specifically designed based on the sequences available in the GenBank database (Supplementary Table S1).

### 2.3 Statistical analysis

The statistical analysis of the data was performed using SPSS software (Version 21.0). An independent samples t-test was used to compare the data between the two groups. Prior to conducting the t-test, the normality of the data was assessed using the Shapiro–Wilk test and Quantile-Quantile plots. The replicate was considered as the experimental unit for analysis. A probability value (*p*-value) below 0.05 was considered statistically significant, indicating a significant difference between the groups.

## 3 Results

### 3.1 Laying performance of layers as affected by NCG supplementation

**Figure 1:**
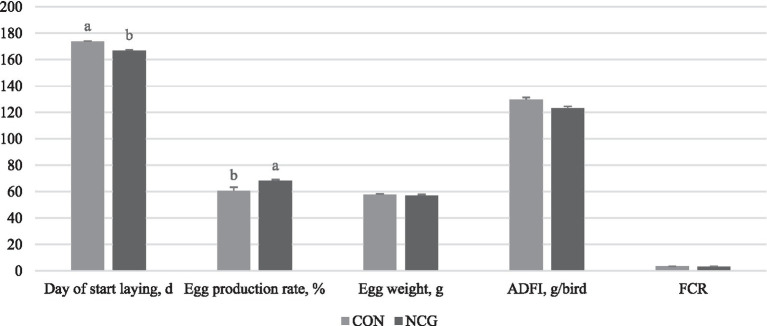
Effects of N-carbamylglutamate supplementation on production performance of layers. ADFI, average daily feed intake; FCR, feed conversion ratio. CON was defined as layers fed with basal diet. NCG was defined as layers fed with basal diet supplemented with 0.12% N-carbamylglutamate.

The findings of the present study are consistent with previous reports by Ma et al. (7, 8, 13), which demonstrated that including NCG in the diet of layers can lead to improvements in production performance. Specifically, our results showed that layers fed with a diet containing NCG exhibited a significant increase in egg production rate (*p* < 0.05) and an advancement in the timing of egg-laying (*p* < 0.05). However, no significant effects were observed on egg weight, ADFI, and FCR (Figure 1; Table 2).

**Table 2:** Effects of dietary NCG supplementation on age at first egg and production performance.

Group	Day of start laying (d)	Egg production rate (%)	Egg weight (g)	ADFI (g/bird)	FCR
CON	173.74 ± 0.39^a^	60.750 ± 2.569^b^	57.725 ± 0.519^a^	129.750 ± 1.707^a^	3.360 ± 0.115^a^
NCG	166.93 ± 0.50^b^	68.182 ± 1.089^a^	56.985 ± 1.007^a^	123.00 ± 1.660^a^	3.170 ± 0.168^a^

ADFI, average daily feed intake; FCR, feed conversion ratio.

Values within a column bearing different superscript letters differ significantly (*p* < 0.05), whereas shared letters indicate no statistical significance.

CON was defined as layers fed with basal diet.

NCG was defined as layers fed with basal diet supplemented with 0.12% N-carbamylglutamate.

### 3.2 Ovarian follicle classification and measurement

Dietary supplementation with 0.12% NCG significantly altered follicular hierarchy in Dagu hens compared to controls (*p* < 0.05). NCG increased SYF count by 32.2% (11.5 vs. 15.2), LYF by 33.3% (5.1 vs. 6.8), and Hierarchical Follicles by 30.2% (4.3 vs. 5.6). No significant differences occurred in SWF or LWF (*p* > 0.05). This shift indicates enhanced transition from early-stage (LWF) to developmentally critical follicles (SYF/LYF) (Table 3).

**Table 3:** Ovarian follicle population in control and NCG-supplemented Dagu hens.

Follicle class	Small white follicles (1–2 mm)	Large white follicles (2–5 mm)	Small yellow follicles (5–8 mm)	Large yellow follicles (8–12 mm)	Hierarchical follicles (>12 mm)
CON	28.3 ± 1.5a	20.6 ± 1.1a	11.5 ± 0.7b	5.1 ± 0.4b	4.3 ± 0.3b
NCG	25.8 ± 1.2a	22.4 ± 0.9a	15.2 ± 0.9a	6.8 ± 0.5a	5.6 ± 0.4a

CON was defined as layers fed with basal diet.

NCG was defined as layers fed with basal diet supplemented with 0.12% N-carbamylglutamate.

### 3.3 Overview of RNA-seq as affected by NCG supplementation

A total of 18 cDNA libraries were constructed from the hypothalamus-pituitary-Ovary of laying hens, and the raw reads of each library is more than 47 million. After filtering to remove low-quality and linker sequences, more than 46 million clean reads remained. The average GC content in each group was approximately 50%. Moreover, the average percentages of Q20 and Q30 bases were higher than 98 and 94%, respectively, as shown in Supplementary Table S2. These parameters strongly affirm the reliability of the sequencing data obtained in this study.

### 3.4 Identification of differentially expressed genes as affected by NCG supplementation

**Figure 2:**
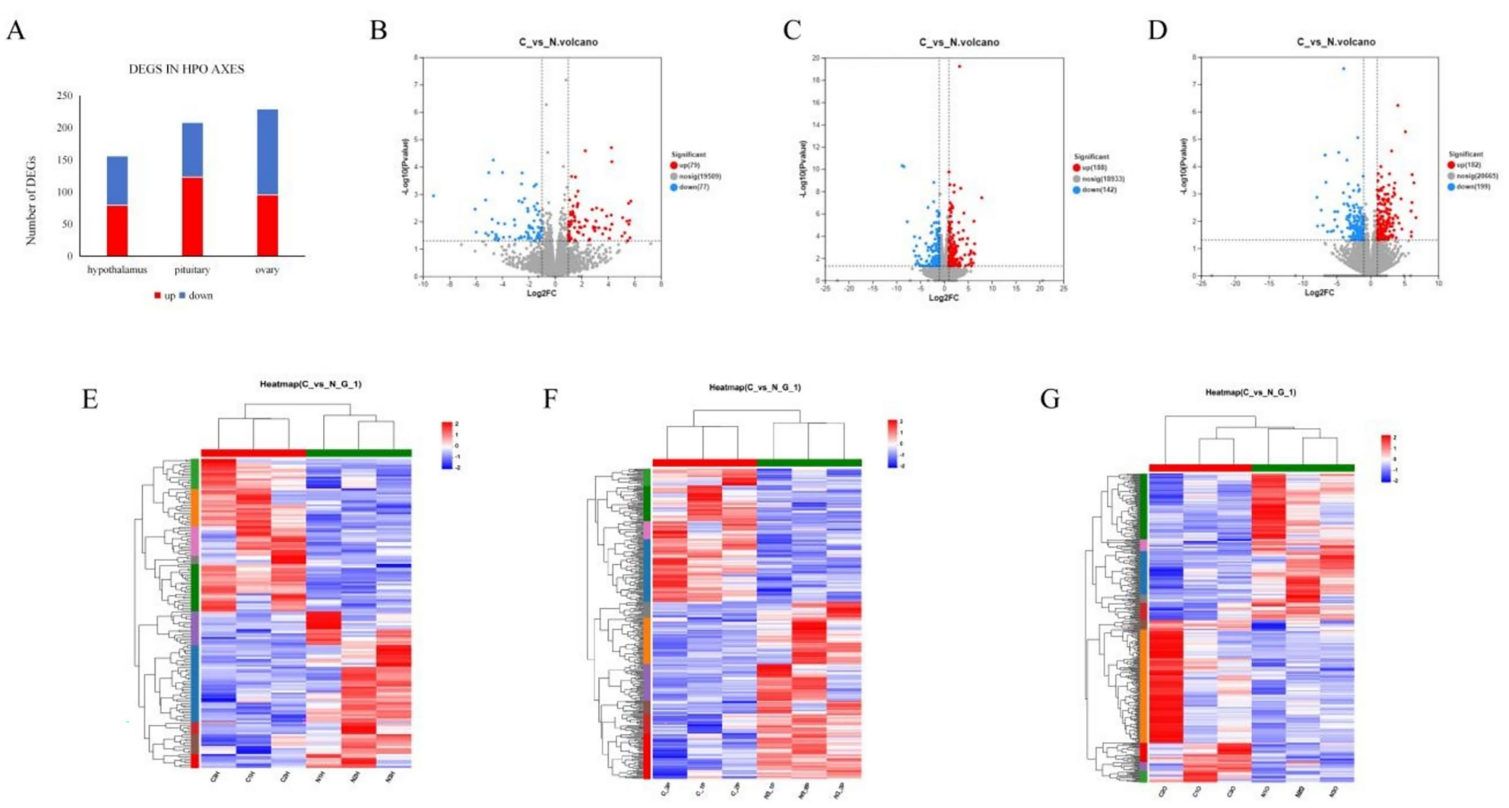
Distribution of DEGs. DEGs identified in 3 tissues **(A)**. Volcano plot of differentially expressed genes in hypothalamic **(B)**, pituitary **(C)**, and ovarian **(D)**; Hierarchical clustering of DEGs in the hypothalamus **(E)**, hierarchical clustering of DEGs in the pituitary **(F)**, hierarchical clustering of DEGs in the ovarian **(G)**.

The RNA-seq results for the 3 tissues revealed the following: in the hypothalamus, a total of 156 DEGs in response to NCG supplementation, among these DEGs, 79 genes were significantly upregulated, while 77 genes were significantly downregulated (*p* < 0.05), Interestingly, among the DEGs, 94 genes were identified as novel genes (Supplementary Data Sheet 1), potentially representing previously uncharacterized genes involved in the response to NCG supplementation; in the pituitary, a total of 208 DEGs in response to NCG supplementation, among these DEGs, 123 genes showed significant upregulation, while 85 genes showed significant downregulation in response to NCG supplementation (*p* < 0.05). Furthermore, out of the identified DEGs, 122 genes were found to be novel genes (Supplementary Data Sheet 2); in the Ovary, a total of 229 DEGs were identified, among these DEGs, 95 were significantly upregulated and 134 were significantly downregulated by NCG supplementation (*p* < 0.05). Furthermore, a total of 152 genes were identified as novel genes (Supplementary Data Sheet 3) (Figure 2A). The volcano plot of DEGs in hypothalamus, pituitary and Ovary is shown in Figures 2B,C; The hierarchical clustering map of TPM depicted the gene expression patterns in the hypothalamus, pituitary and Ovary between CON and TRT, which showed the reliability of the genesets (Figures 2E–G).

To further explore the functional relevance of the DEGs, we conducted screening using the GeneCards database.^4^ As reproduction is of particular interest in this study, we focused on the genes, which are known to be related to reproductive processes. In the hypothalamus, NCG supplementation significantly upregulated the expression of *FSHB* (*p* = 0.003), *GNRH1* (*p* = 0.049) and *POSTN* (*p* = 0.003), and significantly downregulated the expression of *SCNN1B* (*p* = 0.017); In the pituitary, the expression of *POSTN* and *SCNN1B* were significantly upregulated by NCG supplementation (*p* < 0.001), and the expression of *GNRHR* (*p* = 0.019), *COL6A2* (*p* = 0.004), *FN1*(*p* = 0.007)were also significantly upregulated, *MMP* (*p* = 0.012) was downregulated; In the ovary, NCG supplementation led to a significant upregulation in the expression of *POSTN* (*p* = 0.003), while the expression of SCNN1B (*p* = 0.008) was significantly downregulated, as shown in Table 4. These findings suggest that NCG supplementation may exert its effects on the HPO axis, potentially influencing reproductive processes in layers.

**Table 4:** Differentially expressed genes involved in reproductive function from different tissues.

Gene symbol	Description	*p* value	Log_2_FC	Expression level
Hypothalamus				
*FSHB*	Follicle stimulating hormone beta subunit	0.003	3.137	Up
*GNRH1*	Gonadotropin releasing hormone 1	0.049	1.064	Up
*POSTN*	Periostin	0.003	1.676	Up
*SCNN1B*	Sodium channel epithelial 1 beta subunit	0.017	−1.004	Down
Pituitary				
*GNRHR*	Gonadotropin-releasing hormone receptor	0.019	1.357	Up
*POSTN*	Periostin	0.007	1.555	Up
*SCNN1B*	Sodium channel epithelial 1 beta subunit	0.002	1.207	Up
*FN1*	Fibronectin 1	0.007	1.031	Up
Ovary				
*POSTN*	Periostin	0.003	2.348	Up
*SCNN1B*	Sodium channel epithelial 1 beta subunit	0.008	−1.234	Down

### 3.5 Functional enrichment analysis of DEGs

**Figure 3:**
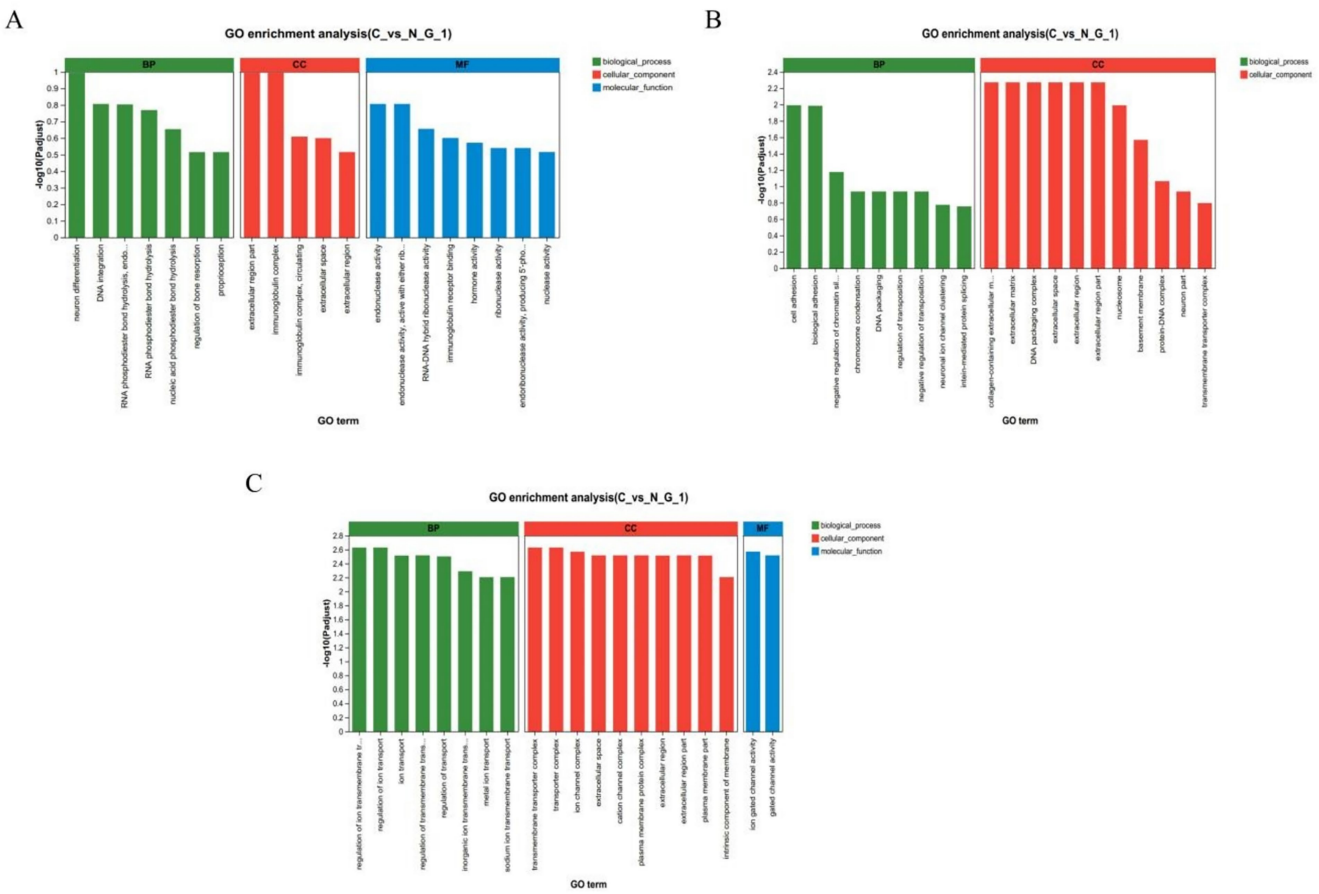
GO enrichment analysis. GO enrichment analysis chart in hypothalamic **(A)**, pituitary **(B)**, and ovarian **(C)**; The horizontal axis represents the GO term, and the vertical axis represents the significance level of enrichment, corresponding to the height of the column, where the smaller the Padjust-The larger the log10 (Padjust) value, the more significantly enriched the GO term. The three colors represent the three major classifications, namely biological processes (BP), cellular components (CC), and molecular functions (MF).

To further elucidate the function of DEGs, we used GO enrichment analysis to annotate DEGs and to study their distribution. In the hypothalamus, GO functional enrichment is concentrated in neuronal differentiation extracellular region part, immunoglobulin complex, endonuclease activity. In the pituitary gland, it is concentrated in the collagen containing extracellular matrix extracellular matrix, DNA packaging complex, extracellular space, extracellular region, cell adhesion, biological adhesion. In the ovaries, it is concentrated in the regulation of ion transmembrane transport regulation of ion transport, transmembrane transporter complex, transporter complex, ion gated channel activity (Figures 3A–C).

**Figure 4:**
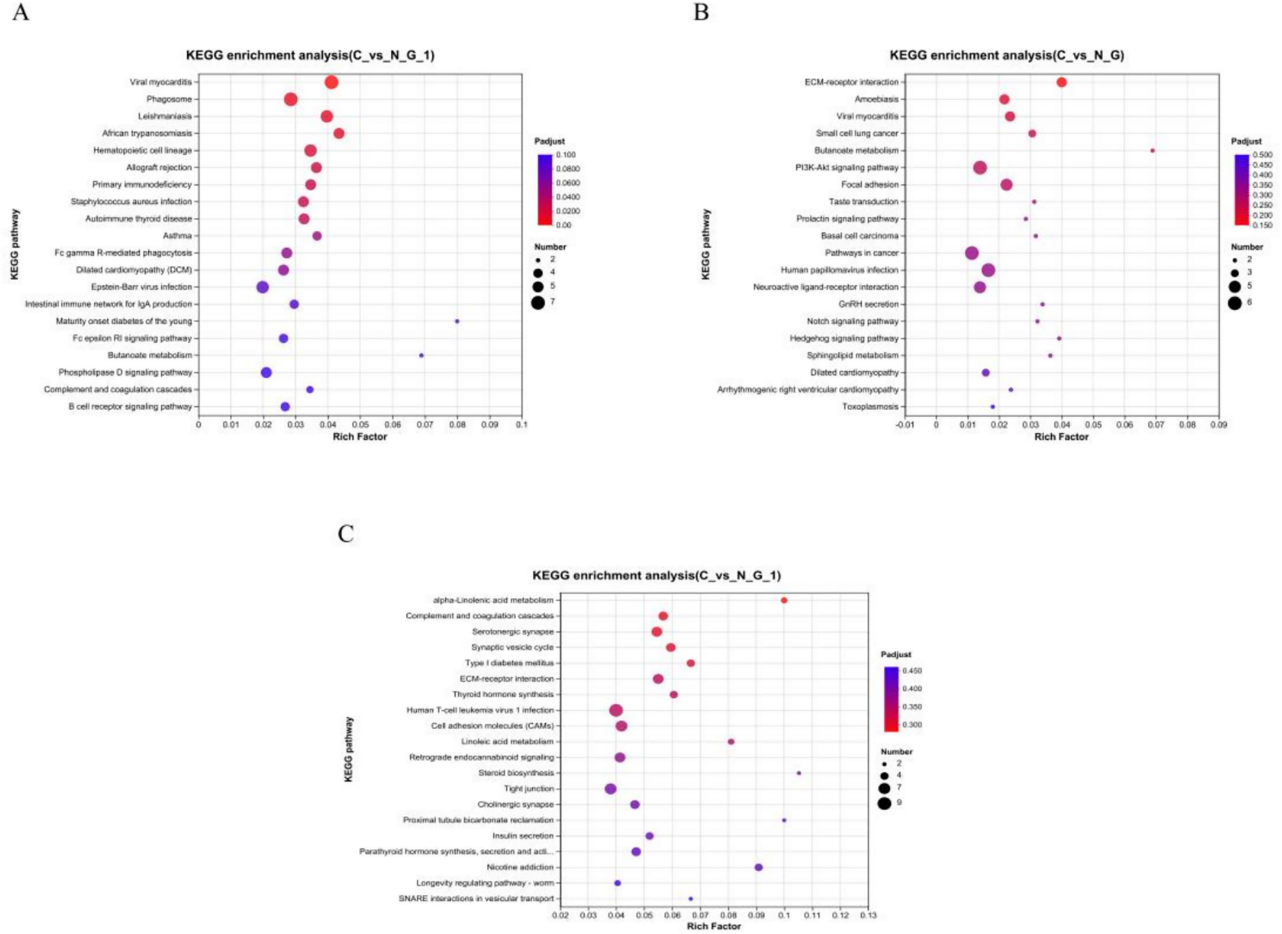
KEGG pathway enrichment analysis. Pathway enrichment analysis of differentially expressed genes (DEGs) in hypothalamic **(A)**, pituitary **(B)**, and ovarian **(C)**. The x-axis represents rich factor (rich factor = number of DEGs enriched in the pathway/number of all genes in the background gene set). The y-axis represents the enriched pathway. Color represents enrichment significance, and the size of the bubble represents the number of DEGs enriched in the pathway.

To further identify the major biochemical, metabolic, and signal transduction pathways of the DEGs, we performed a KEGG pathway enrichment analysis. A total of 29, 17, and 22 KEGG pathways were enriched in the hypothalamus, pituitary, and ovary, respectively (Supplementary Data Sheets 4−6). In the hypothalamus, two pathways, namely butanoate metabolism (*p* = 0.014) and calcium signaling pathway (*p* = 0.034), showed significant enrichment, in the pituitary, butanoate metabolism (*p* = 0.009), ECM-receptor interaction (*p* = 0.001), PI3K-Akt signaling pathway (*p* = 0.018), taste transduction (*p* = 0.039), the prolactin signaling pathway (*p* = 0.046), neuroactive ligand-receptor interaction (*p* = 0.031), GnRH secretion (*p* = 0.034), the notch signaling pathway (*p* = 0.037), and the hedgehog signaling pathway (*p* = 0.026), showed significant enrichment; in the ovary, alpha-Linolenic acid metabolism (*p* = 0.013), ECM-receptor interaction (*p* = 0.010), thyroid hormone synthesis (*p* = 0.024), linoleic acid metabolism (*p* = 0.023), retrograde endocannabinoid signaling (*p* = 0.034), steroid biosynthesis (*p* = 0.039), insulin secretion (*p* = 0.039), taste transduction (*p* = 0.005), and neuroactive ligand-receptor interaction (*p* = 0.003), showed significant enrichment. These findings suggest that these pathways are potentially involved in the molecular processes and signaling networks associated with 3 tissue function (Table 5; Figure 4).

**Table 5:** Functional annotation of differentially expressed genes by KEGG database for different tissues.

Tissues	Pathway ID	Description	*p* value	Enriched genes (part)
Hypothalamus	map00650	Butanoate metabolism	0.014	*Metazoa_SRP*
	map04020	Calcium signaling pathway	0.034	*P2RX4*
Hypophysis	map00650	Butanoate metabolism	0.009	*Metazoa_SRP*
	map04151	PI3K-Akt signaling pathway	0.018	*COL6A2* *FN1*
	map04742	Taste transduction	0.039	*GABRA5* *HTR1B*
	map04917	Prolactin signaling pathway	0.046	*Prl* *SOCS3*
	map04080	Neuroactive ligand-receptor interaction	0.031	*GABRA5* *P2RY8*
	map04929	GnRH secretion	0.034	*KCNN1* *TRPC5*
	map04330	Notch signaling pathway	0.037	*HES5*
	map04340	Hedgehog signaling pathway	0.026	*DHH* *HHIP*
Ovary	map00592	alpha-Linolenic acid metabolism	0.013	*PLA2G4F*
	map04512	ECM-receptor interaction	0.009	*COL9A2* *FN1*
	map04918	Thyroid hormone synthesis	0.024	*TTR* *TPO*
	map00591	Linoleic acid metabolism	0.023	*PLA2G4F*
	map04723	Retrograde endocannabinoid signaling	0.034	*GABRA3* *CACNA1B*
	map00100	Steroid biosynthesis	0.039	*DHCR7* *CYP24A1*
	map04911	Insulin secretion	0.039	*GCG* *STX1A*
	map04080	Neuroactive ligand-receptor interaction	0.003	*SSTR3* *GLRB*
	map04742	Taste transduction	0.005	*SCNN1B* *GABRA3*

### 3.6 qRT-PCR validation

**Figure 5:**
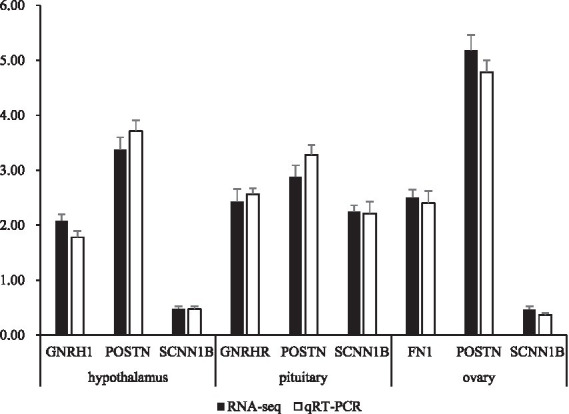
Comparison gene expression between qRT-PCR and RNA-seq. The data represent the logarithm of the gene expression level in the TRT group compared to its expression level in the CON group. Values represent the means of 5 replicates per group (*n* = 5).

To validate the accuracy of the RNA-seq results, 7 DEGs were selected for qRT-PCR. genes *FSHB*, *GNRH1*, *POSTN*, *SCNN1B*, *GNRHR*, *COL6A2* and *FN1* were included. Taking b-actin and GAPDH as reference genes, the expression levels of the genes were consistent with the RNA-seq results (Figure 5).

## 4 Discussion

The time of starting egg-laying in layers is closely linked to the degree of maturation of their sexual organs, as highlighted by Shi et al. (14). Early maturation of sexual organs indicates the initiation of the animals’ productive phase, and an earlier onset of maturation is associated with higher overall production potential. In the case of layers, the advancement of starting egg-laying reflects the precocity of their sexual organs. Previous studies have demonstrated that the supplementation of NCG can enhance the maturation of sexual organs in birds. For instance, Ma et al. (6) fed roosters with a diet containing NCG and observed improvements in parameters related to seminiferous tubules, as well as increased levels of serum gonadotropin-releasing hormone and testosterone, along with the development of secondary sexual characteristics. Furthermore, a transcriptomics analysis conducted on the testicles of these roosters revealed the regulation of genes involved in gonadal function maintenance, steroid hormone biosynthesis, and metabolism under NCG supplementation. Additionally, Ma et al. (8) reported that NCG supplementation upregulated the expression of genes associated with vitamin A metabolism, nutrient transport, protein synthesis, and calcium transport in the ovaries of layers, resulting in increased egg production rate and improved egg quality. Therefore, the advancement of starting egg-laying observed with NCG supplementation may be attributed to its ability to promote the development of sexual organs.

Notably, yellow follicles constitute the fundamental basis of egg-laying potential, with both their quantity and quality exerting direct effects on reproductive performance, as highlighted by Brady et al. (15, 16). Previous research has demonstrated that the supplementation of NCG can promote the development of ovarian follicles by enhancing angiogenesis in the ovaries of layers, as reported by Ma et al. (13). Avian egg production is determined by both the number of follicles available for ovulation and the oviduct’s capacity to transform oocytes into hard-shelled eggs. In addition to environmental and metabolic factors, follicular growth and development are regulated by a complex interplay of endocrine, paracrine, and autocrine factors, including gonadotropins, sex steroid hormones, and growth factors. In poultry, reproductive endocrine activity and ovarian function are tightly controlled by the HPO axis (17). The hypothalamus regulates reproduction by releasing neurohormones to the pituitary gland. In response, the pituitary synthesizes and secretes gonadotropins that act on the gonads, stimulating both gonadal development (spermatogenesis and oogenesis) and the secretion of gonadal steroid hormones. Within the ovary, granulosa cells serve as the primary site of estrogen production while also providing endocrine signals to other tissues. These ovarian cells support oocyte growth and development, preparing for the LH surge that triggers the physiological cascade of ovulation. This process involves promoting meiosis, steroidogenesis, follicular development, cumulus cell expansion, luteinization, and progesterone production, ultimately leading to oocyte maturation (18, 19). Therefore, the observed increase in SYF, LYF and egg production rate in this study may be attributed to NCG’s regulatory effects on follicular development through the HPO axis.

Furthermore, egg weight is an important economic parameter that influences consumer acceptance of eggs. Additionally, the FCR is a measure of nutrient absorption efficiency, and a lower FCR indicates improved production performance (1). However, in the current study, NCG supplementation did not have a significant effect on egg weight or FCR. This suggests that the improvement in laying performance observed with NCG supplementation may not be attributed to enhanced nutrient absorption. Other mechanisms, such as the regulation of reproductive hormones, ovarian follicle development, or other physiological processes, may be involved in the observed improvement in laying performance in layers. Further research is needed to elucidate the underlying mechanisms of NCG supplementation on egg production and performance in layers.

Based on the findings of this study, it is evident that NCG supplementation positively impacts the laying performance of layers. Since the reproductive performance of layers is regulated by the HPO axis, it is crucial to investigate the specific effects of NCG supplementation on this axis. To gain further insights into the endocrine mechanisms underlying the improvement in laying performance induced by NCG supplementation, a comprehensive transcriptomics analysis was conducted on the hypothalamus, pituitary, and ovary. This analysis aimed to uncover the molecular and genetic changes associated with NCG supplementation and shed light on the specific pathways and genes involved in regulating the laying performance of layers.

As reproduction is of particular interest in this study, we focused on the differentially expressed genes *FSHB* and *GNRH1*in the hypothalamus, *GnRHR* in the pituitary, and *POSTN* and *SCNN1B*, which are known to be involved in the reproductive process.

*FSHB*, which encodes the beta subunit of follicle-stimulating hormone (FSH), plays a pivotal role in ovarian follicle production (20). FSH, secreted by the pituitary gland and regulated by the hypothalamus, consists of an alpha subunit and the hormone-specific beta subunit.^5^ Abnormal expression of *FSHB* has been linked to hypogonadism, a condition characterized by impaired reproductive function (21). *FSHB* is widely recognized as a key gene involved in stimulating folliculogenesis (22). Studies comparing the sequences of genes related to follicular development in different goat breeds have identified variations in the amino acid sequences of FSHB, suggesting its potential role in determining reproductive capacity (23). In zebrafish, knockout experiments targeting *FSHB* have demonstrated that it is crucial for ovary growth and the activation of follicles, as its suppression resulted in inhibited ovary growth and complete blockade of follicle activation (24, 25).

The *GNRH1* gene encodes a family of gonadotropin-releasing hormone peptides, including gonadoliberin-1 and GnRH-associated peptide 1, which play important roles in reproductive processes. Gonadoliberin-1 specifically stimulates the release of luteinizing hormone (LH) and FSH, crucial hormones involved in reproduction. Abnormal expression of GNRH1 has been linked to hypogonadism, a condition characterized by impaired reproductive function (26). Due to its relevance to fertility, *GNRH1* is frequently examined in animal experiments (27, 28). In a study by Fan et al. (29) involving female Bama miniature pigs, supplementation with estradiol valerate resulted in an increase in the age of puberty and a decrease in estrus ratio. These changes were associated with the downregulation of the *GNRH1* gene in the hypothalamus.

The *GNRHR* gene encodes the receptor for type 1 gonadotropin-releasing hormone (GnRH). Upon binding of GnRH, the receptor interacts with G-proteins, leading to the activation of a phosphatidylinositol-calcium second messenger system. This activation ultimately results in the release of LH and FSH, key hormones involved in reproductive processes. Abnormal expression of *GNRHR* has been identified as a cause of hypogonadotropic hypogonadism, a condition characterized by reduced gonadal functio (30). A specific case study reported homozygous partial loss-of-function mutations in the *GNRHR* gene associated with constitutional delay of growth and puberty (31). Additionally, Lovell et al. (32) observed an increase in pituitary mRNA levels of *GNRHR* during the preovulatory surge in layers, indicating its involvement in the regulation of reproductive processes. The upregulation of *GNRHR* expression in the pituitary following NCG supplementation in this study suggests a potential mechanism for the improvement in reproductive performance observed in layers.

The HPO axis is the core neuroendocrine system that regulates the reproductive function of poultry, and *POSTN* (periodin) and *SCNN1B* (epithelial sodium channel *β* subunit) as common differentially expressed genes in this axis may affect the reproductive performance of chickens through multi tissue synergy.

*POSTN* (Periostin) gene encodes an extracellular matrix protein, which belongs to the osteoblast specific factor 2 (OSF-2) (33), family and plays an important role in tissue development, damage repair and reproductive regulation. High levels of Periostin have been detected in human ovaries (34). The protein functions as a ligand for AVB 3 and AVB 5 integrins to support the adhesion and migration of ovarian epithelial cells (35), In chicken, the postn gene consists of 23 exons and encodes a polypeptide of 841 amino acids (36), Previous studies have demonstrated that in mesenchymal cells undergoing differentiation, the expression of postn is significantly increased in response to the growth factor signaling of bone morphogenetic protein 2/4 (BMP 2/4) and transforming growth factor B (TGF-b) (37), BMP 2/4 and tgf-β genes are both involved in the development of hen ovary (36, 38, 39), and there are research results supporting the postn gene the polymorphisms were associated with egg production and egg weight or body weight (40). The upregulation of *POSTN* expression observed in the HPO axis in response to NCG supplementation in this study suggests that NCG may have a positive impact on the regulation of gonadal development and function.

*SCNN1B* gene (full name: sodium channel epithelial 1 beta subunit), which encodes an important component of epithelial sodium channel (ENaC), is involved in regulating intracellular and extracellular sodium ion balance, fluid homeostasis and transmembrane signaling (41, 42), Previous studies have shown that scnn1a, scnn1b, and scnn1g are highly expressed in the uterus during eggshell formation (43, 44), While scnn1g expression increased rapidly during the eggshell formation stage. Genes that are highly specifically expressed in specific tissues may contribute to their development and function (45). Genetic studies have shown that the scnn1 gene family has tissue-specific expression and is significantly correlated with eggshell traits and age at the start of production (46), In this study, scnn1b gene was significantly expressed in three tissues of HPO axis, and the previous research results of the research group showed that the age at the beginning of production and eggshell strength of NCG supplemented group were better than those of the control group (47), It shows that the addition of NCG can regulate the expression of scnn1 gene family of HPO axis and positively regulate the laying performance of laying hens.

The functional annotation of DEGs in three tissues using the KEGG database revealed, the butyric acid metabolism pathway is significantly enriched in the hypothalamus and pituitary gland, the ECM receptor interaction pathway is significantly enriched in the pituitary gland and ovary, and the taste transduction pathway is significantly enriched in the hypothalamus and ovary, These pathways may play important roles in the regulatory mechanisms influenced by NCG supplementation in the HPO axis.

The involvement of butanoate metabolism pathway in the regulation of reproductive processes has been observed in previous studies. For instance, the production of butyrate, a metabolite generated during butanoate metabolism, has been shown to induce the acetylation of histone H3K9, leading to the activation of steroidogenesis in ovarian granulosa cells. This activation occurs through the involvement of pathways such as PPARγ and PGC1α (48).

Furthermore, Zhang et al. (49) conducted an RNA-seq analysis to investigate the variation of DEGs in the hypothalamus between polytocous and monotocous sheep during the luteal phase. They discovered that DEGs from the hypothalamus of polytocous sheep were significantly enriched in the butanoate metabolism pathway. These findings suggest the potential involvement of butanoate metabolism in the regulation of reproductive processes and its relevance to the observed effects of NCG supplementation on the hypothalamus and pituitary in the present study.

The extracellular matrix (*ECM*) plays a pivotal role in regulating various cellular processes essential for follicle growth and oocyte maturation. It exerts both promoting and inhibitory effects on cellular activities, including proliferation, differentiation, and survival. Proteins encoded by *ECM* genes have been found to have a significant impact on the morphology, survival, proliferation, and steroidogenesis of granulosa cells, follicles, and whole ovaries in culture, as demonstrated by Berkholtz et al. (50). Their study elucidated the influence of these ECM proteins on the cellular dynamics within the ovary, highlighting their crucial involvement in ovarian function. The ECM provides structural support and organization to ovarian follicles, contributing to the regulation of cellular behavior and interactions within the ovarian microenvironment. By modulating cellular processes, the ECM plays a vital role in the proper development and maturation of follicles and oocytes.

Taste receptors, which are commonly associated with the perception of flavors, have been found to be expressed in various sexual organ (51). However, our understanding of the effects of taste transduction on reproduction is still limited. A recent study by Semplici et al. (52) shed light on the potential role of bitter taste receptors in the female reproductive system. Their findings indicated that the activation of taste receptors was associated with improved fertility. These results suggest a possible link between taste transduction and reproductive processes, highlighting the need for further research in this area.

## 5 Conclusion

In summary, our findings provide compelling evidence that NCG modulates laying performance in hens by regulating key genes (including FSHB, GNRHR, and RSPO1) within the HPO axis, offering new insights into its beneficial effects on follicular development and egg production efficiency. Building upon these discoveries, future research should focus on: optimizing NCG dosage regimens and administration protocols to establish precision nutritional strategies; employing multi-omics approaches to elucidate the critical signaling pathways through which NCG regulates the reproductive axis; and identifying novel molecular targets for poultry breeding programs. These advancements will provide both theoretical foundations and technical support for developing innovative feed additives and implementing sustainable, high-efficiency poultry production systems.

## Data Availability

The data presented in the study is deposited in the https://www.ncbi.nlm.nih.gov/ repository, accession number: PRJNA1331643.

## Footnotes

^1^ https://arriveguidelines.org/arrive-guidelines/experimental-animals

^2^ https://mart.ensembl.org/Gallus_gallus/Info/index

^3^ http://www.genome.jp/kegg/

^4^ https://www.genecards.org/

^5^ https://en.wikipedia.org/wiki/FSHB
