# Supplementary material for: N-carbamylglutamate supplementation improves laying performance of layers by regulating hypothalamic-pituitary-ovarian axis
Source: Front Vet Sci. 2025 Oct 2;12:1668137. doi: 10.3389/fvets.2025.1668137 (PMC12529935; doi:10.3389/fvets.2025.1668137)
Supplement: Supplementary file 7 [file Table_1.docx]

鸡的，FSHB，GNRH1，POSTN，SCNN1B，GNRHR，COL6A2，FN1

Table S1. Primer information for target gene amplification

| Gene | Primer Information | Fragment Length | Reference Gene Sequence |
| --- | --- | --- | --- |
| *FSHB* | F: 5′ GCACTCTACTAGAATACAGGATGA 3′ | 172 | NM_204257.2 |
|  | R: 5′ AAGCAGTATCCTGAGCACCAC 3′ |  |  |
| *GNRH1* | F: 5′ GGCAATCTGCTTGGCTCAAC 3′ | 197 | NM_001080877.1 |
|  | R: 5′ CGATCAGGCTTGCCATGGTT 3′ |  |  |
| *GNRHR* | F: 5′ GCAGCTGCAGGAGGGG 3′ | 199 | NM_001012609.1 |
|  | R: 5′ GCACAAAGAGCACGAAGGTG 3′ |  |  |
| *POSTN* | F: 5′ AGTCCATTTGGAAGAAGCACC 3′ | 146 | NM_001030541.2 |
|  | R: 5′ AGACCACGCTGTTTTCTTGT 3′ |  |  |
| *SCNN1B* | F: 5′ CTTCGAGACCAACCAGAGCG 3′ | 113 | XM_040647613.2 |
|  | R: 5′ GGTGGCTGCACAGCTTCA 3′ |  |  |
| *COL6A2* | F: 5′ CTAGGGGCTCTTTGTGGCAG 3′ | 249 | NM_205348.4 |
|  | R: 5′ CTGGGCTACTGCAAGCTCTGT 3′ |  |  |
| *FN1* | F: 5′ TTGGAGAGCAGTGGCAGAAG 3′ | 192 | NM_001198712.2 |
|  | R: 5′ GGCAGTTGACGTTGGTGTTT 3′ |  |  |
