# Supplementary material for: N-carbamylglutamate supplementation improves laying performance of layers by regulating hypothalamic-pituitary-ovarian axis
Source: Front Vet Sci. 2025 Oct 2;12:1668137. doi: 10.3389/fvets.2025.1668137 (PMC12529935; doi:10.3389/fvets.2025.1668137)
Supplement: Supplementary file 8 [file Table_2.docx]

| **Table S2.** Quality analysis of sequencing data in different tissues | | | | | | | |  |
| --- | --- | --- | --- | --- | --- | --- | --- | --- |
| Tissues | | Raw reads^c^ | Clean reads^d^ | Error rate, % | Q20^e^, % | Q30^f^, % | GC content^g^, % | Total mapped^h^, % |
| Hypothalamus | |  |  |  |  |  |  |  |
| C1^a^ | | 57913154 | 57250628 | 0.0248 | 98.06 | 94.34 | 49.02 | 92.68 |
| C2^a^ | | 58450592 | 57750606 | 0.0249 | 98.02 | 94.23 | 49.12 | 92.89 |
| C3^a^ | | 55550614 | 54878524 | 0.0249 | 98.01 | 94.26 | 49.52 | 92.67 |
| N1^b^ | | 57817136 | 57043430 | 0.0254 | 97.81 | 93.75 | 49.41 | 92.91 |
| N2^b^ | | 57742788 | 57073410 | 0.0248 | 98.05 | 94.34 | 49.49 | 92.66 |
| N3^b^ | | 53888004 | 53276166 | 0.0249 | 98.04 | 94.29 | 49.16 | 93.05 |
| Pituitary | |  |  |  |  |  |  |  |
| C1 | | 47464250 | 46965332 | 0.0247 | 98.12 | 94.52 | 49.49 | 92.70 |
| C2 | | 64706680 | 63943454 | 0.0248 | 98.06 | 94.42 | 50.82 | 92.01 |
| C3 | | 58443366 | 57669212 | 0.0253 | 97.85 | 93.90 | 50.18 | 92.03 |
| N1 | | 52544122 | 51967840 | 0.0248 | 98.06 | 94.36 | 50.17 | 92.70 |
| N2 | | 58277310 | 57611098 | 0.0247 | 98.10 | 94.50 | 50.61 | 91.99 |
| N3 | | 57376852 | 56767950 | 0.0246 | 98.15 | 94.59 | 50.06 | 92.07 |
| Ovary | |  |  |  |  |  |  |  |
| C1 | | 65103550 | 64459418 | 0.0246 | 98.18 | 94.61 | 50.36 | 93.79 |
| C2 | | 59219342 | 58575244 | 0.0248 | 98.08 | 94.39 | 49.84 | 93.13 |
| C3 | | 55492378 | 54938822 | 0.0248 | 98.10 | 94.37 | 49.71 | 94.02 |
| N1 | | 60492906 | 59865734 | 0.0247 | 98.14 | 94.51 | 50.39 | 92.72 |
| N2 | | 59411836 | 58807444 | 0.0246 | 98.17 | 94.61 | 49.90 | 93.58 |
| N3 | | 63171020 | 62471442 | 0.0248 | 98.09 | 94.42 | 50.33 | 93.58 |
| ^a^C1, C2, C3 are samples from the group of layers fed with basal diet. | | | | | | | |  |
| ^b^N1, N2, N3 are samples from the group of layers fed with 0.12% N-carbamylglutamate containing diet. | | | | | | | |  |
| ^c^The statistics of the number of sequencing sequences generated from library construction. | | | | | | | |  |
| ^d^The statistics of the number sequencing sequences filtered by sequencing data. | | | | | | | |  |
| ^e^The percent of sequenced bases that have a predicted quality score of 20. | | | | | | | |  |
| ^f^The percent of sequenced bases that have a predicted quality score of 30. | | | | | | | |  |
| ^g^The dependence between fragment counts (read coverage). | | | | | | | |  |
| ^h^The ratio of the clean reads mapped to the reference genome. | | | | | | | |  |
